# Metabolic and psychiatric effects of acyl coenzyme A binding protein (ACBP)/diazepam binding inhibitor (DBI)

**DOI:** 10.1038/s41419-020-2716-5

**Published:** 2020-07-06

**Authors:** Adrien Joseph, Stéphanie Moriceau, Valentina Sica, Gerasimos Anagnostopoulos, Jonathan Pol, Isabelle Martins, Antoine Lafarge, Maria Chiara Maiuri, Marion Leboyer, Josephine Loftus, Frank Bellivier, Raoul Belzeaux, Fabrice Berna, Bruno Etain, Delphine Capdevielle, Philippe Courtet, Caroline Dubertret, Julien Dubreucq, D’. Amato Thierry, Guillaume Fond, Sebastien Gard, Pierre-Michel Llorca, Jasmina Mallet, David Misdrahi, Emilie Olié, Christine Passerieux, Mircea Polosan, Paul Roux, Ludovic Samalin, Franck Schürhoff, Raymond Schwan, the FACE-SZ and FACE-BD (FondaMental Academic Centers of Expertise, for Schizophrenia and for Bipolar Disorder) Groups, Christophe Magnan, Franck Oury, José M. Bravo-San Pedro, Guido Kroemer

**Affiliations:** 1 Centre de Recherche des Cordeliers, Equipe labellisée par la Ligue Contre le Cancer, Université de Paris, Sorbonne Université, Inserm U1138, Paris, France; 2 Metabolomics and Cell Biology Platforms, Institut Gustave Roussy, Villejuif, France; 3 Faculté de Médecine, Université de Paris Saclay, Kremlin Bicetre, France; 4 INSERM U1151, Institut Necker Enfants-Malades (INEM), Université Paris Descartes-Sorbonne-Paris Cité, Paris, France; 5 Cell Biology Group, Department of Experimental and Health Sciences, Pompeu Fabra University (UPF), Barcelona, Spain; 6 Gustave Roussy Comprehensive Cancer Institute, Villejuif, France; 7 Fondation FondaMental, Créteil, France; 8 Université Paris Est Créteil, Inserm U955, IMRB, Laboratoire Neuro-Psychiatrie translationnelle, F-94010 Créteil, France; 9 AP-HP, HU Henri Mondor, Departement Medico-Universitaire de Psychiatrie et d’Addictologie (DMU ADAPT), Federation Hospitalo-Universitaire de Médecine de Precision (FHU IMPACT), F-94010 Créteil, France; 10 Fondation FondaMental Créteil, Créteil, France; 11 Pôle de Psychiatrie, Centre Hospitalier Princesse Grace, Monaco, France; 12 AP-HP, GH Saint-Louis—Lariboisière—Fernand Widal, Pôle Neurosciences Tête et Cou, INSERM UMRS 1144, University Paris Diderot, Paris, France; 13 Pôle de Psychiatrie, Assistance Publique Hôpitaux de Marseille, Marseille, France; 14 INT-UMR7289, CNRS Aix-Marseille Université, Marseille, France; 15 Hôpitaux Universitaires de Strasbourg, Université de Strasbourg, INSERM U1114, Fédération de Médecine Translationnelle de Strasbourg, Strasbourg, France; 16 Service Universitaire de Psychiatrie Adulte, Hôpital la Colombière, CHRU Montpellier, Université Montpellier 1, Inserm 1061, Montpellier, France; 17 Department of Emergency Psychiatry and Acute Care, Lapeyronie Hospital, CHU Montpellier, Montpellier, France; 18 PSNREC, Univ Montpellier, INSERM, CHU Montpellier, Montpellier, France; 19 AP-HP, Groupe Hospitalo-Universitaire Nord, DMU ESPRIT, Service de Psychiatrie et Addictologie. Hopital Louis Mourier, Colombes, Inserm U1266, Faculté de Médecine, Université de Paris, Paris, France; 20 Centre Référent de Réhabilitation Psychosociale et de Remédiation Cognitive (C3R), CH, Alpes Isère, France; 21 INSERM U1028, CNRS UMR5292, Centre de Recherche en Neurosciences de Lyon, Université Claude Bernard Lyon 1, Equipe PSYR2, Centre Hospitalier Le Vinatier, Pole Est, 69678 Bron Cedex, France; 22 AP-HM, Aix-Marseille University, School of Medicine—La Timone Medical Campus, EA 3279, Marseille, France; 23 EReSS—Health Service Research and Quality of Life Center, 13005 Marseille, France; 24 Centre Expert Troubles Bipolaires, Service de Psychiatrie Adulte, Hôpital Charles-Perrens, Bordeaux, France; 25 CHU Clermont-Ferrand, Department of Psychiatry, University of Clermont Auvergne, Clermont-Ferrand, France; 26 Service Universitaire de Psychiatrie d’Adultes, Centre Hospitalier de Versailles, Le Chesnay, Université Paris-Saclay, UVSQ, Inserm, CESP, Team “DevPsy”, 94807 Villejuif, France; 27 Université Grenoble Alpes, CHU de Grenoble et des Alpes, Grenoble Institut des Neurosciences (GIN) Inserm U 1216, Grenoble, France; 28 Université de Lorraine, CHRU de Nancy et Pôle de Psychiatrie et Psychologie Clinique, Centre Psychothérapique de Nancy, Nancy, France; 29 Université de Paris, BFA, UMR 8251, CNRS, Paris, France; 30 University Complutense of Madrid. Faculty of Medicine. Department of Physiology, Madrid, Spain; 31 Pôle de Biologie, Hôpital Européen Georges Pompidou, AP-HP, Paris, France; 32 Suzhou Institute for Systems Medicine, Chinese Academy of Medical Sciences, Suzhou, China; 33 Karolinska Institute, Department of Women’s and Children’s Health, Karolinska University Hospital, Stockholm, Sweden

**Keywords:** Endocrine system and metabolic diseases, Psychiatric disorders

## Abstract

Acyl coenzyme A binding protein (ACBP), also known as diazepam binding inhibitor (DBI) is a multifunctional protein with an intracellular action (as ACBP), as well as with an extracellular role (as DBI). The plasma levels of soluble ACBP/DBI are elevated in human obesity and reduced in anorexia nervosa. Accumulating evidence indicates that genetic or antibody-mediated neutralization of ACBP/DBI has anorexigenic effects, thus inhibiting food intake and inducing lipo-catabolic reactions in mice. A number of anorexiants have been withdrawn from clinical development because of their side effects including an increase in depression and suicide. For this reason, we investigated the psychiatric impact of ACBP/DBI in mouse models and patient cohorts. Intravenously (i.v.) injected ACBP/DBI protein conserved its orexigenic function when the protein was mutated to abolish acyl coenzyme A binding, but lost its appetite-stimulatory effect in mice bearing a mutation in the γ2 subunit of the γ-aminobutyric acid (GABA) A receptor (GABA_A_R). ACBP/DBI neutralization by intraperitoneal (i.p.) injection of a specific mAb blunted excessive food intake in starved and leptin-deficient mice, but not in ghrelin-treated animals. Neither i.v. nor i.p. injected anti-ACBP/DBI antibody affected the behavior of mice in the dark–light box and open-field test. In contrast, ACBP/DBI increased immobility in the forced swim test, while anti-ACBP/DBI antibody counteracted this sign of depression. In patients diagnosed with therapy-resistant bipolar disorder or schizophrenia, ACBP/DBI similarly correlated with body mass index (BMI), not with the psychiatric diagnosis. Patients with high levels of ACBP/DBI were at risk of dyslipidemia and this effect was independent from BMI, as indicated by multivariate analysis. In summary, it appears that ACBP/DBI neutralization has no negative impact on mood and that human depression is not associated with alterations in ACBP/DBI concentrations.

## Introduction

Acyl coenzyme A (CoA) binding protein (ACBP) has been identified as an ubiquitously expressed 86 amino acid polypeptide that binds medium-sized (C_14_–C_22_) acyl CoA chains in the cytoplasm of multiple (if not all) cell types^1^. In addition, this protein acts as an “endozepine” and displaces benzodiazepines such as tritium-labeled diazepam from its receptors, hence acting as diazepam binding inhibitor (DBI)^1^. There are two benzodiazepine receptors, the peripheral receptor, a mitochondrion-located translocator protein (TSPO), and a central receptor, which is the γ-aminobutyric acid (GABA) A receptor (GABA_A_R), the major inhibitory neurotransmitter receptor in the central nervous system. Full length ACBP/DBI displaces diazepam from both TSPO and GABA_A_R. In the central nervous system, ACBP/DBI produced by astrocytes and other cell types can be subjected to endoproteolytic cleavage to generate neuropeptides such as triakontatetraneuropeptide (residues 17–50) that acts as a selective ligand of TSPO and octadecaneuropeptide (residues 33–50) that acts as an allosteric modulator of GABA_A_R activity^1^.

Recently, we reported that ACBP/DBI plasma concentration is abnormally high in obese individuals, correlating with the fact that the periumbilical fat from obese persons expresses high levels of ACBP/DBI mRNA that diminish upon dietary intervention^2^. Similarly, in mice, obesity induced by a high-fat diet or a genetic deficiency of leptin results into increased expression of ACBP/DBI mRNA and protein in the liver and in adipose tissue, accompanied by an increase in circulating ACBP/DBI protein levels^2^. Conversely, anorexia nervosa is associated with a reduction in ACBP/DBI plasma level^2,3^. A prior study had shown that in patients with acute inflammatory disease, ACBP/DBI plasma levels increase, positively correlating with tumor necrosis factor-α (TNFα) levels^4^. Intriguingly, obesity is coupled to a state of chronic inflammation in which TNFα is elevated, contributing to the development of insulin-resistant (type-2) diabetes^5,6^. This points to a relationship between metabolic inflammation and the elevation of mediators such as ACBP/DBI and TNFα.

Experiments in mice revealed that intravenous (i.v.) injection of ACBP/DBI protein or transgenic expression of ACBP/DBI in the liver caused hyperphagy and weight gain. Conversely, neutralization of ACBP/DBI by an inducible whole-body knockout or intraperitoneal (i.p.) injection of neutralizing antibodies had anorexigenic effects, reducing food intake and lipo-anabolic reactions, while increasing lipo-catabolism (such as lipolysis and fatty acid oxidation), thus reducing weight gain in the context of a high-fat diet or leptin deficiency or enhancing weight loss upon a switch from a high-fat diet to a normal diet^2^. These findings, combined with the fact that ACBP/DBI, an evolutionarily ancient gene/protein, can stimulate sporulation in unicellular yeast species^7,8^ and in slime moldsan^9^, pharyngeal pumping in nematodes^8^, and mouse hook movement (the equivalent of mastication) in flies^10^ let us to postulate that ACBP/DBI is the elusive phylogenetically conserved appetite stimulator or “hunger factor”^11,12^.

Eating disorders such as anorexia nervosa and morbid obesity are metabolic diseases with a psychiatric component. Importantly, prototypic psychiatric diseases including treatment-resistant depression and severe schizophrenia are coupled to major derangements in appetite and body weight and often lead to a state of metabolic syndrome that negatively affects life expectancy^13–15^. Obviously, the GABAergic system composed by GABA and its receptors plays a major role in the central nervous system^16,17^ as well as in the regulation of metabolism^18^ and inflammation^19^.

Intrigued by these premises, we decided to investigate the possible impact of ACBP/DBI on psychiatric conditions. For this, we addressed the questions as to whether ACBP/DBI stimulates appetite through its binding to acyl CoA or an action on GABA_A_R and whether ACBP/DBI affects the behavior of mice upon its artificial elevation or neutralization in peripheral tissues. We also measured ACBP/DBI concentration in the plasma of psychiatric patients to understand its potential impact on mental vs. metabolic disease.

## Materials and methods

### Mouse experiments

Eight- to ten-week-old male C57BL/6 mice, Wild-type (WT, Envigo, Gannat, France and Janvier, Le Genest-Saint-Islen, France), B6.Cg-Lep^ob^/J *ob/ob* mice, S/B6.V-LEP+*/Ob* (JAX^™^ Mice Strain, Charles River Laboratory, Lentilly, France) or Gabrg2^tm1Wul^/J, containing the point mutation F77I in the gamma-aminobutyric acid (GABA) A receptor γ2 subunit^20^ (JAX^™^ Mice Strain, Charles River Laboratory, Lentilly, France) were bred and maintained according to the FELASA guidelines and local guidelines from the Animal Experimental Ethics Committee (#04447.02, #2315-2015101617138161v1, #8530-2017011216394941v2, #10862-2017080217568517v3, #25032, 19144-201805041255279v2, France).

### Treatments

Mice were housed in a temperature-controlled environment with 12 h light/dark cycles and received normal diet and water ad libitum. Mice were subjected to 24 h starvation (Unfed), injected intraperitoneally or intravenously and cumulative food intake was analyzed. The mAb 7A antibody against ACBP/DBI or the isotype IgG2a control were used in vivo (5 µg/g body weight (BW), i.p, in total volume 200 μL) (Fred Hutch Antibody Technology, Seattle, WA, USA). Recombinant mouse ACBP/DBI (i.v., in total volume of 200 μL, 0.5 mg/kg BW) (recACBP/DBI, from Institute of Psychiatry and Neuroscience of Paris, France) or the vehicle control (phosphate-buffered saline) were used in vivo. Moreover, two mutant forms of mouse recombinant ACBP/DBI were used in which two conserved residues were substituted (Y29F and K33A), reducing the affinity of ACBP/DBI for the acyl-CoAs^21^. Recombinant mouse Ghrelin (purchased by Merk Millipore) was administered by i.p. injection at 10 µg/25 g BW.

### Food intake analysis

Food intake was monitored as previously described^2^. In brief, food was removed 2 h prior to experimentation followed by individual housing and acclimatization in individual cages. Different treatments were administered and the accumulated food intake was monitored.

### Light-to-dark transition test (D/LT)

Test based on the innate aversion of rodents to brightly illuminated areas and on their spontaneous exploratory behavior in response to the stressor that light represents^22^. The test apparatus consists of a dark, safe compartment and an illuminated, aversive one (43 × 43 cm chamber). The lit compartment was brightly illuminated with an 8 W fluorescent tube (1000 lx). Naive mice were placed individually in the testing chamber in the middle of the dark area facing away from the doorway to the light compartment. Mice were tested for 10 min, and four parameters were recorded: time spent in the lit compartment, the number of transitions between compartments, the speed of the mice and the distance spent in the lit compartment indices of anxiety-related behavior and exploratory activity. Behavior was scored using an infrared light beam activity monitor using actiMot2 Software (PhenoMaster Software, TSE) and it was statistically analyzed using Prism program.

### Open-field test (OFT)

Test takes advantage of the aversion of rodents to brightly lit areas^22^. Each mouse is placed in the center of the OFT chamber (43 × 43 cm chamber) and allowed to explore for 30 min. Mice were monitored throughout each test session by infrared light beam activity monitor using actiMot2 Software (PhenoMaster Software, TSE). The overall motor activity was quantified as the total distance travelled (ambulation). Anxiety was quantified by measuring the time and distance spent in the center versus periphery of the open-field chamber. Behavior was scored using an infrared light beam activity monitor using actiMot2 Software (PhenoMaster Software, TSE) and it was statistically analyzed using Prism program.

### Forced swim test (FST)

Test based on the observation that rodents, after initial escape-oriented movements, develop an immobile posture when placed in an inescapable stressful situation^23^. Each mouse is placed in a cylinder (height: 25 cm and diameter: 10 cm) filled with water (23–25 °C). Mice were tested for 5 min, and the time spent immobile (behavioral despair) was quantified.

### ACBP/DBI detection in human plasma samples

Plasma ACBP/DBI levels were measured in two different cohorts of bipolar and schizophrenic patients, by means of the KA0532 ACBP (Human) ELISA kit. The subjects (*n* = 271) were participants of the FACE-BD and FACE-SZ studies^13–15^. Dyslipidaemia and type 2 diabetes were extracted from patient’s medical history. Hypertension was defined as systolic blood pressure ≥ 140 and/or diastolic blood pressure ≥ 90 mmHg. Abdominal obesity was defined as waist circumference ≥ 94 cm or 37 in. (male) or ≥80 cm or 31.5 in. (female). Metabolic syndrome was defined according to the International Diabetes Foundation definition^24^.

### Statistical analysis

Data are reported as Box and whisker plots (mean, first and third quartiles, and maximum and minimum values). The number of independent data points (*n*) is indicated in the corresponding graphs or in the legends. For statistical analyses, *p* values were calculated by two-way ANOVA, one-way ANOVA with Tukey’s multiple comparisons test or two-tailed unpaired Student’s *t* test as indicated (Prism version 7, GraphPad Software). Differences were considered statistically significant when *p* values *(*p* < 0.05), **(*p* < 0.01), ***(*p* < 0.001), and n.s. = not significant (*p* > 0.05). For the analysis of human samples, means (±standard deviation or standard error of the mean) were compared with two-tailed unpaired Student’s *t* test and Pearson’s correlation coefficients with their 95% confidence interval were calculated. A generalized linear model was constructed to calculate odds ratios between ACBP/DBI (per 1 ng/mL increase) and categorical metabolic variables in a univariate model and in a multivariate model incorporating body mass index (BMI).

## Results

### Appetite stimulation by ACBP/DBI in mice through an action on GABA_A_ receptors

**Fig. 1:**
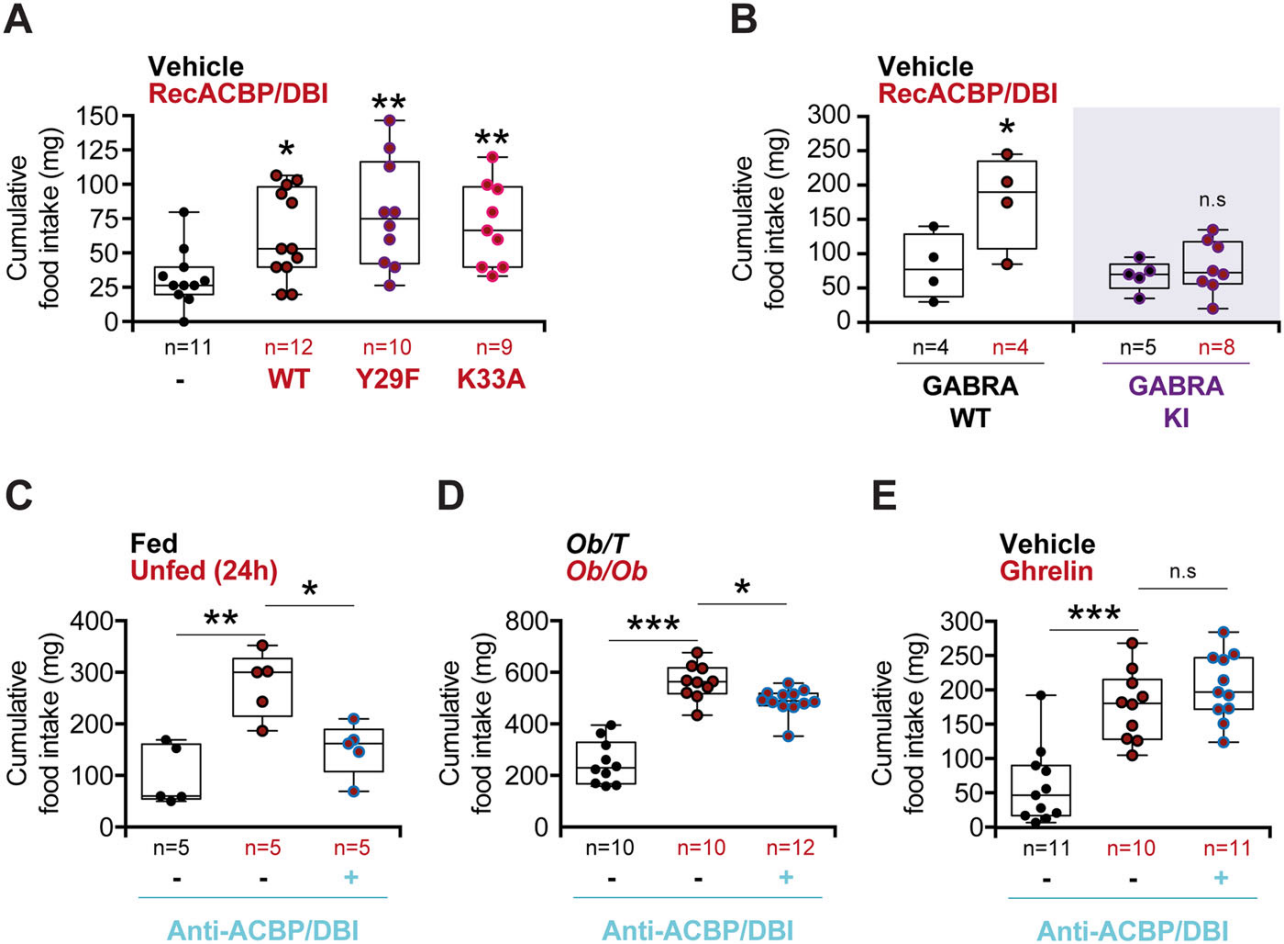
Analysis of feeding behavior modulated by ACBP/DBI. **a** Cumulative food intake was measured after 60 min in WT mice injected with recombinant ACBP/DBI (recACBP/DBI) protein, its mutant forms Y29F or K33A (i.v., 0.5 mg/kg BW) or a vehicle control (**a**), in GABRA WT or GABRA knock-in (KI) mice upon recACBP/DBI i.v. injection (**b**), in WT mice after 24 h of starvation (Unfed) (**c**), in obese *Ob/Ob* or lean *Ob/T* mice (**d**), and in WT mice after Ghrelin injection (i.p., 10 µg/25 g BW) (**e**), all of them either alone or in combination with the i.p injection of an antibody against ACBP/DBI (anti-ACBP/DBI, i.p., 5 µg/g BW). Quantitative results are reported as box and whisker plots (mean, first and third quartiles, and maximum and minimum values). For statistical analyses, *p* values were calculated by two-way ANOVA (**b**) or one-way ANOVA with Tukey’s multiple comparisons test (**a**, **c**–**e**). Differences were considered statistically significant when *p* values *(*p* < 0.05), **(*p* < 0.01), ***(*p* < 0.001) and n.s. not significant (*p* > 0.05).

As indicated by its dual name, ACBP/DBI has two fundamentally distinct functions, as a protein that binds acyl coenzyme A (CoA) and as a protein that binds to GABA_A_R. The interaction with acyl-CoA is reduced by 3 orders of magnitude upon mutation of tyrosine residue 29 to phenylalanine, Y29F, or mutation of lysine residue 33 to alanine, K33A (Supplementary Fig. S1a)^21,25^. I.v. injection of such mutated Y29F or K33A ACBP/DBI recombinant proteins induced a similar hyperphagic response as did the WT protein (Fig. 1a), indicating that appetite stimulation by ACBP/DBI does not rely exclusively on the binding of acyl-CoA-related metabolites. The action of ACBP/DBI on GABA_A_ receptor is lost in mice in which the γ2 subunit bears a point mutation substituting the phenylalanine residue 77 to isoleucine, F77I) (Supplementary Fig. S1b)^20,26^. Mice bearing this knockin (KI) mutation failed to mount a hyperphagic response upon injection of WT ACBP/DBI in conditions in which age- and sex-matched WT control mice did increase their food intake (Fig. 1b), indicating that ACBP/DBI indeed acts on GABA_A_ receptors to stimulate appetite.

Of note, i.p. injection of a neutralizing ACBP/DBI-specific monoclonal antibody (mAb) was able to inhibit food intake in mice that had been rendered hyperphagic (Supplementary Fig. S1c) by a 24-h starvation period (Fig. 1c). Similarly, anti-ACBP/DBI mAb reduced food intake in mice homozygous for the *Lep*^ob^ mutation (often referred to as *ob*/*ob* mice) that are rendered hyperphagic due to a mutation in the gene encoding for the appetite inhibitor leptin (Fig. 1d). In contrast, ACBP/DBI neutralization was unable to interfere with hyperphagy induced by ghrelin injection (Fig. 1e), indicating that anti-ACBP/DBI mAb has a specific rather than general effect on food intake. Thus, the possibility that anti-ACBP/DBI mAb would simply induce a general lethargy that compromises food intake can be excluded.

### Effects of ACBP/DBI on the behavior of mice

**Fig. 2:**
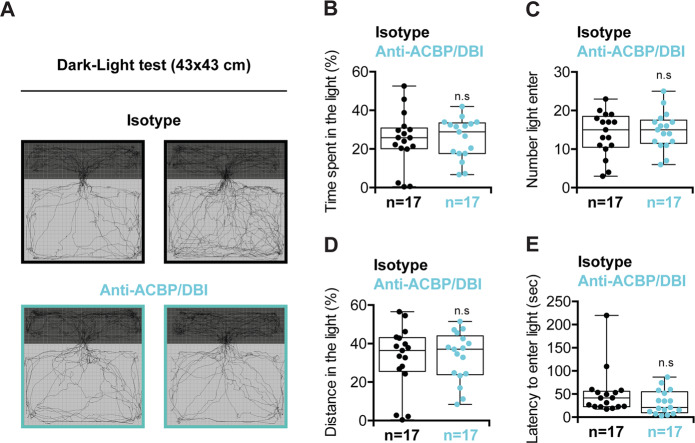
Dark–light test. **a** Examples of trajectories during the test by untreated (isotype) (upper panels) or anti-ACBP/DBI-treated mice (lower panels). **b** Percentage of time spent in the light (%), **c** number of accesses to light, **d** percentage of distance travelled in the light, and **e** latency to enter light in seconds were measured for 10 min. Quantitative results are reported as Box and whisker plots (mean, first and third quartiles, and maximum and minimum values) (*n* = 17). Symbols indicate statistical (Student’s *t* test) comparisons with isotype control (n.s not significant).

**Fig. 3:**
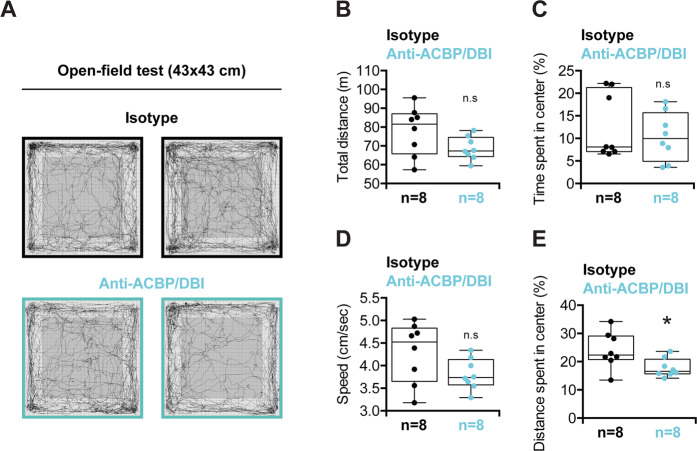
Open-field test. **a** Examples of trajectories during the test by untreated (isotype) (upper panels) or anti-ACBP/DBI-treated mice (lower panels). **b** Total distance, **c** percentage of time spent in center (%), **d** speed, and **e** percentage of distance spent in center were measured during 30 min. Quantitative results are reported as Box and whisker plots (mean, first and third quartiles, and maximum and minimum values) (*n* = 8). Symbols indicate statistical (Student’s *t* test) comparisons with isotype control (n.s not significant and *p < 0.05).

Pharmacological agents acting on GABA_A_ receptors (which include anesthetics, barbiturates, benzodiazepines, and zolpidem) have major effects on human behavior^16,27^, and several appetite-inhibitory agents have been rejected or withdrawn by either the FDA or EMA (or both) due to an increase in depression and suicide^28,29^, prompting us to assess the behavioral effects of ACBP/DBI neutralization in mouse models. In the light–dark box test, which measures unconditioned anxiety and that accurately reflects the anxiolytic effects of benzodiazepines^30,31^, mice receiving the neutralizing anti-ACBP/DBI antibody exhibited a similar behavior as control mice injected with an isotype control antibody (Fig. 2). Similarly, ACBP/DBI injection had no impact on this behavioral test (Supplementary Fig. S2). The open-field test, which measures general locomotor activity levels, anxiety, and willingness to explore, is known to be sensitive to benzodiazepines^32–34^. ACBP/DBI neutralization had no major effects on the open-field test, except for a longer distance spent in the center of the box, suggesting a mild anxiolytic activity for the anti-ACBP/DBI antibody (Fig. 3). However, recombinant ACBP/DBI did not affect the open-field test (Supplementary Fig. S3).

**Fig. 4:**
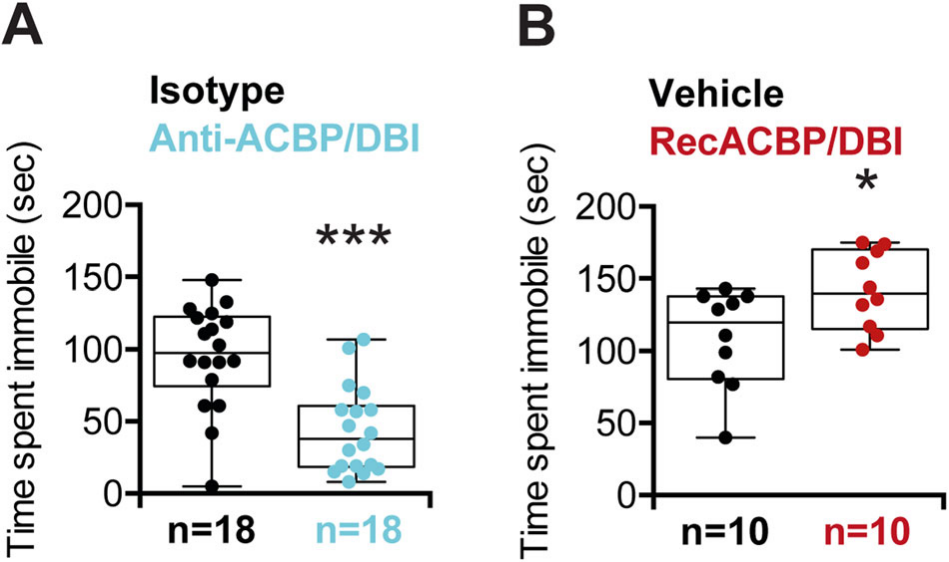
Immobility time in the forced swim test. Time spent immobile in seconds after anti-ACBP/DBI (*n* = 18) (**b**) or recACBP/DBI (*n* = 10) (**c**) treatments were measured for 5 min. Quantitative results are reported as Box and whisker plots (mean, first and third quartiles, and maximum and minimum values). Symbols indicate statistical (Student’s *t* test) comparisons with controls (*p < 0.05 and ***p < 0.001).

Next, we took advantage of the Porsolt forced swim test (also called “behavioral despair test”) (Supplementary Fig. S4a), which is used to detect a depression-like behavior, reflected by a premature switch from swimming to immobile floating^23^. Benzodiazepines are well known to enhance the immobile behavior in this test in a dose-dependent fashion^35,36^. Of note, the anti-ACBP/DBI antibody reduced the immobile behavior of mice (Fig. 4a), while injection of recombinant ACBP/DBI protein enhanced the floating behavior (Fig. 4b), in line with the interpretation that ACBP/DBI neutralization has an antidepressant effect. Of note, the effects of ACBP/DBI on depression were lost when the protein was mutated to suppress its acyl CoA binding ability (Supplementary Fig. S4b).

### Plasma ACBP/DBI levels in patients diagnosed with severe depression or schizophrenia

**Fig. 5:**
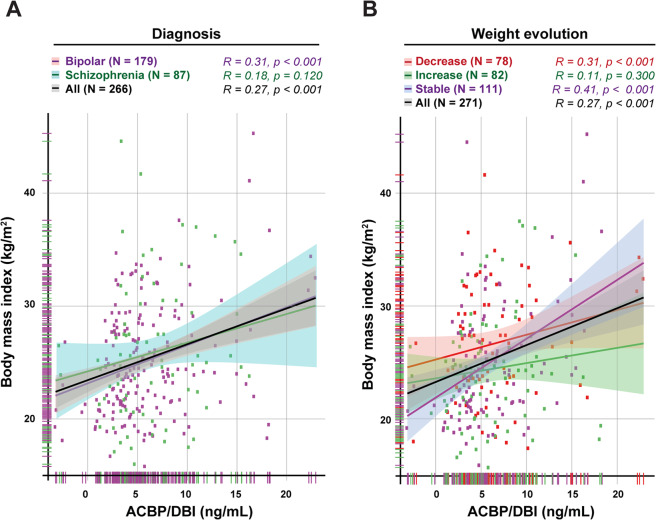
No impact of pychiatric disease or weight evolution on the correlation between ACBP/DBI plasma concentration and body mass index. Scatter plot with regression line between ACBP/DBI (ng/mL) and body mass index (kg/m^2^) in bipolar and schizophrenic patients (**a**) and patients who lose weight (≥5%), gain weight (≥5%), or remain stable (variations < 5%) (**b**). Pearson’s correlation coefficient (*R*) and their *p* value are shown on top of each panel.

In the next step, we measured the circulating ACBP/DBI concentration in patients with bipolar disorder or schizophrenia. No difference in the plasma ACBP/DBI levels was detectable between depressive and schizophrenic patients (Fig. 5a, Supplementary Fig. S5a). In both groups, ACBP/DBI levels similarly correlated with the BMI, body weight or waist circumference (Supplementary Fig. S5d), as previously described for a series of patients with eating disorders including anorexia nervosa and morbid obesity^2^. ACBP/DBI concentrations did not correlate with subsequent weight variations, meaning that there were no significant variations in ACBP/DBI concentration between patients with minor weight oscillations (by <5), significant weight loss (≥5%) or weight gain (≥5%) within the 6 months following the ACBP/DBI measurement (Fig. 5b, Supplementary Fig. S5b).

**Fig. 6:**
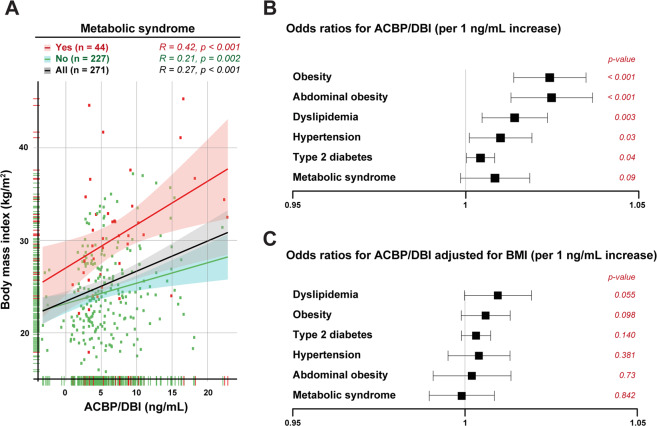
Impact of metabolic syndrome on ACBP/DBI levels. No impact of pychiatric disease or weight evolution on the correlation between ACBP/DBI plasma concentration and body mass index. Scatter plot with regression line between ACBP/DBI (ng/mL) and body mass index (kg/m^2^) in patients with or without metabolic syndrome. Pearson’s correlation coefficient (*R*) and their *p* value are shown on top of each panel. Forest plots representing odds ratios for the association between ACBP/DBI (per 1ng/mL increase) and obesity, abdominal obesity, dyslipidemia, hypertension, type 2 diabetes, and metabolic syndrome in univariate analysis (**b**) and after adjustment for body mass index (**c**).

Nonetheless, patients with metabolic syndrome tended to exhibit a higher ACBP/DBI concentration than patients without metabolic syndrome (*p* = 0.097) (Fig. 6a, Supplementary Fig. S5c). However, multivariate analysis indicated that ACBP/DBI was not associated with metabolic syndrome, independently of BMI. In contrast, variations in ACBP/DBI plasma levels were associated with dyslipidemia (*p* = 0.003) (but not with diabetes nor arterial hypertension), and this association was independent of BMI (*p* = 0.055) (Fig. 6b, c).

Altogether, these results suggest that ACBP/DBI levels are well correlated with BMI, irrespective of their psychiatric diagnosis, do not predict later changes in BMI, yet are associated with dyslipidemia.

## Discussion

ACBP/DBI is a phylogenetically ancient protein that stimulates appetite and lipo-anabolism in animals, ranging from nematodes and insects to rodents^8,12^. It is also elevated in human obesity but reduced in anorexia nervosa^2^. For this reason, neutralization of ACBP/DBI by suitable antibodies might constitute a valid strategy for combating obesity and its co-morbidities^12,37,38^. Given the fact that several anorexigenic drugs have been withdrawn from the clinics due to their psychiatric side effects^27,28^, we evaluated the behavioral effect of ACBP/DBI and ACBP/DBI neutralizing antibodies in rodent models and attempted to establish a correlation between mental disease and circulating ACBP/DBI concentrations in psychiatric patients.

Mouse experiments detailed in this paper revealed that the orexigenic effect of systemically (i.v.) injected ACBP/DBI protein did not rely on its interaction with acyl-CoA but apparently involved an action on GABA_A_R, alerting about the possibility that ACBP/DBI might indeed affect GABA-regulated mood control. However, at odds with this possibility, neither the systemic (i.v.) injection of ACBP/DBI nor the systemic (i.p.) administration of a neutralizing ACBP/DBI antibody did affect the behavior of mice in the light–dark test and in the open-field tests. In contrast, ACBP/DBI injection caused a “depression-like” behavior in the forced swim test, meaning that the mice ceased active swimming and switched toward passive floating earlier than sham-injected mice. Conversely, neutralization of ACBP/DBI resulted into an “antidepressant” effect, prolonging the active combat of mice for survival. The effects of ACBP/DBI on depression depend on its acyl CoA binding ability, while induction of hyperphagy by ACBP/DBI did not require acyl CoA binding. These discrepant findings underscore that (some of) the metabolic and mood-modulating effect of ACBP/DBI can be uncoupled from each other.

Mice that are constitutively knockout for ACBP/DBI (meaning that the gene is even expressed during embryogenesis) exhibit a stereotyped self-grooming behavior, reduced social interest, but normal social recognition^39^, pointing to a minor behavioral phenotype. In contrast, we have not noted any evident changes in mouse behavior after the inducible knockout of ACBP/DBI in adult mice^2^, suggesting that these effects might be linked to neurodevelopment. Intracerebroventricular administration of recombinant ACBP/DBI or that of ACBP/DBI-derived neuropeptides induces proconflict behavior^40^, stimulates anxiety^41^, and reduces food intake^42^, causing a loss in bodyweight in long-term experiments^43^. When microinjected into the swallowing pattern generator located in the nucleus tractus solitarius, the octadecaneuropeptide derived from ACBP/DBI inhibits the swallowing reflex^42^. Of note, the anorexigenic effects of octadecaneuropetide do not depend on an action on TSPO or GABA_A_R but rather on a G protein coupled receptor^44,45^. Thus, the central (i.c.v.) injection of ACBP/DBI causes GABA_A_R-independent anorexigenic effects that are diametrically opposed to the GABA_A_R-dependent orexigenic effects observed after its peripheral (i.v.) administration^2^. Of note, it appears plausible that i.v. administered ACBP/DBI mediates its effects through an action on peripheral metabolism, causing a hypoglycemic response that then activates orexigenic neurons in the hypothalamus. Indeed, artificial maintenance of glucose concentrations by a glucose clamp prevents the activation of such orexigenic neurons as well as the hyperphagic response of mice^2^.

Obviously, it will be interesting to investigate the impact of ACBP/DBI on the expression of its receptors (in particular GABAAR subunits and the mitochondrial TSPO protein), the expression level of other neuroendocrine factors, as well as bioenergetic parameters in multiple different peripheral and central nervous tissue to understand the full range of its physiological effects. Thus a single-cell multi-omics approach (including but not limited to transcriptomics, proteomics and metabolomics) should be envisaged in the future to explore the effects of ACBP/DBI in further detail.

In line with the idea that the peripheral pool of ACBP/DBI has little impact on mental operations, we did not observe any difference between schizophrenic and bipolar patients with respect to their plasma ACBP/DBI concentrations, which however strongly correlated with BMI in both groups. The levels of ACBP/DBI concentrations measured at diagnosis did not allow predicting the subsequent trajectory of BMI (gain, loss, or stability) and rather correlated with the actual state of the BMI. However, a high ACBP/DBI plasma concentration constitutes a risk factor for dyslipidemia, independently from BMI, as indicated by multivariate analysis. This result strongly pleads in favor of a role of ACBP/DBI in metabolism that is more important than its putative role in mental disease.

As a final note, it appears important that ACBP/DBI neutralization, which might constitute a novel treatment for obesity and its comorbidities such as type-2 diabetes and non-alcoholic steatohepatitis, has no unwarranted (depression- or anxiety-inducing) effects on mice. This preclinical finding may facilitate the development of a novel type of antiobesity medication that targets ACBP/DBI or its interaction with peripheral GABA_A_R.

## Supplementary information

Supplementary Figure Legends

Supplementary Figure S1

Supplementary Figure S2

Supplementary Figure S3

Supplementary Figure S4

Supplementary Figure S5
