# Supplementary material for: Metabolic and psychiatric effects of acyl coenzyme A binding protein (ACBP)/diazepam binding inhibitor (DBI)
Source: Cell Death Dis. 2020 Jul 6;11(7):502. doi: 10.1038/s41419-020-2716-5 (PMC7338362; doi:10.1038/s41419-020-2716-5)
Supplement: Supplementary file 1 — Supplementary Figure Legends [file 41419_2020_2716_MOESM1_ESM.docx]

**Supplementary Figure Legends**

**Supplementary Fig. S1.**

(A) Protein sequence features between the WT and mutant ACBP/DBI protein. (B) Scheme of GABRA activity in Gabrg2^tm1Wul^/J mice. (C) Types of food intake induction used in figure 1.

**Supplementary Fig. S2.**

(A) Percentage of time spent in the light (%), (B) number of accesses to light, (C) percentage of distance in the light and (D) latency to enter light in seconds were measured for 10 min in untreated (isotype) (n = 10) or recombinant ACBP/DBI (recACBP/DBI)-treated (n = 9) mice. Quantitative results are reported as Box and whisker plots (mean, first and third quartiles, and maximum and minimum values). Symbols indicate statistical (Student’s *t* test) comparisons with isotype control (n.s. = not significant).

**Supplementary Fig. S3.**

**Open-field test.** (B) Total distance, (C) percentage of time spent in center (%), (D) speed and (E) percentage of distance spent in center were measured for 30 min in untreated (vehicle) (n = 10) or recombinant ACBP/DBI (recACBP/DBI)-treated (n = 9) mice. Quantitative results are reported as Box and whisker plots (mean, first and third quartiles, and maximum and minimum values). Symbols indicate statistical (Student’s *t* test) comparisons with isotype control (n.s.: not significant).

**Supplementary Fig. S4.** Schematic characteristic behaviors in the forced swim test.

**Supplementary Fig. S5.**

**Plasma ACBP/DBI was measured in cohorts of patients diagnosed with bipolar disorders or schizophrenia (A-C).** Comparisons between ACBP/DBI levels were performed in based on (A) diagnosis (bipolar disorders, n = 179 or schizophrenia; n = 87), (B) weight oscillations (decrease, n = 78: increase n = 82; stable n = 111) or (C) presence or not metabolic syndrome (yes, n = 44 or no, n = 227). Data are shown as means ± standard error of the mean, compared with two-tailed unpaired Student’s t-test (A, C) or one way-ANOVA followed by Tukey's multiple comparisons test (B). n.s. = not significant (p>0.05). **Correlation plot between baseline variables of bipolar and schizophrenic patients (D)**. Positive correlations are displayed in blue and negative correlations in red color. Color intensity and the size of the circle are proportional to the correlation coefficients. Only statistically significant correlations are shown.
