# Supplementary figures and images for: Metabolic and psychiatric effects of acyl coenzyme A binding protein (ACBP)/diazepam binding inhibitor (DBI)
Source: Cell Death Dis. 2020 Jul 6;11(7):502. doi: 10.1038/s41419-020-2716-5 (PMC7338362; doi:10.1038/s41419-020-2716-5)

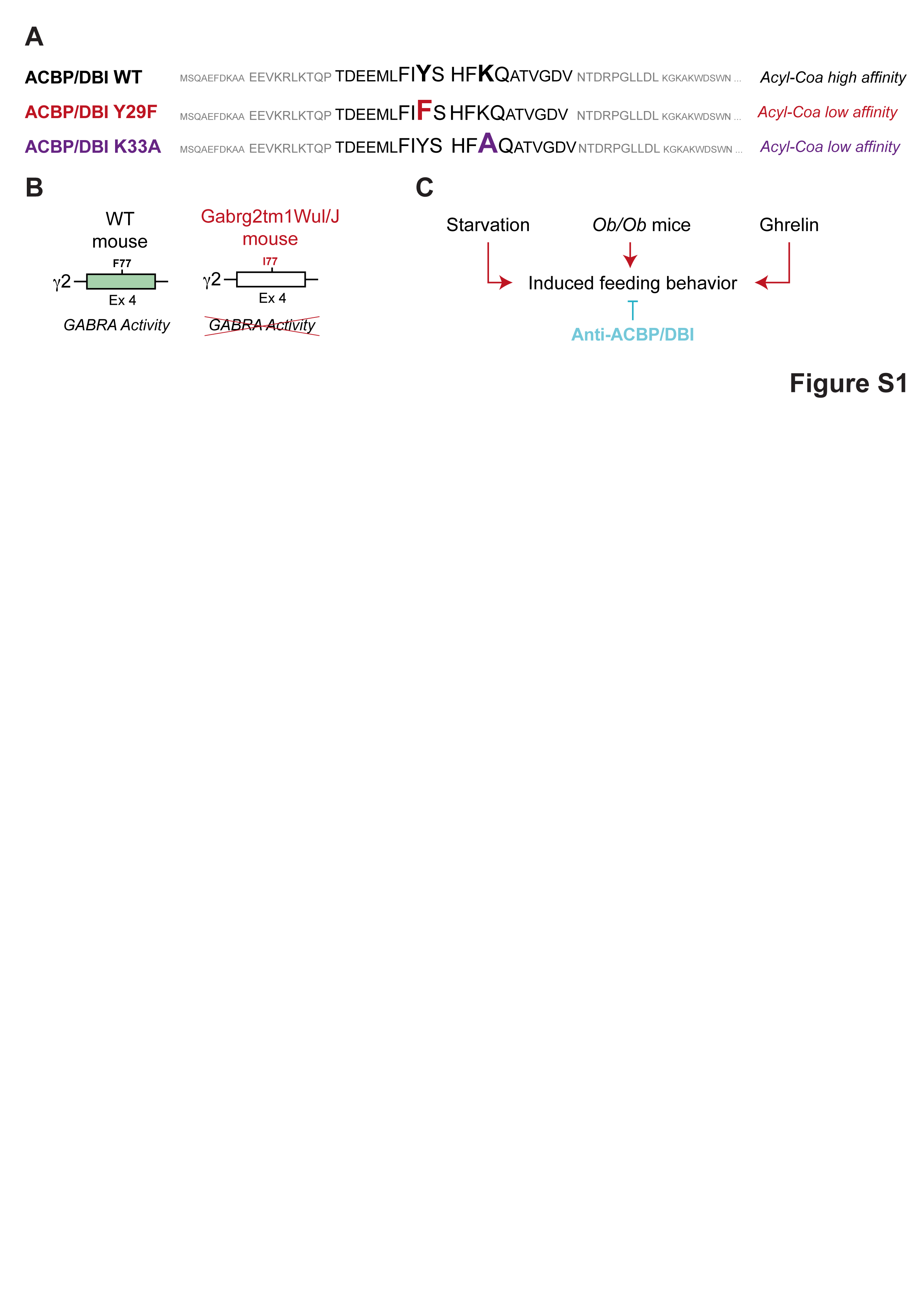

Supplement: Supplementary file 2 — Supplementary Figure S1 [file 41419_2020_2716_MOESM2_ESM.tif]

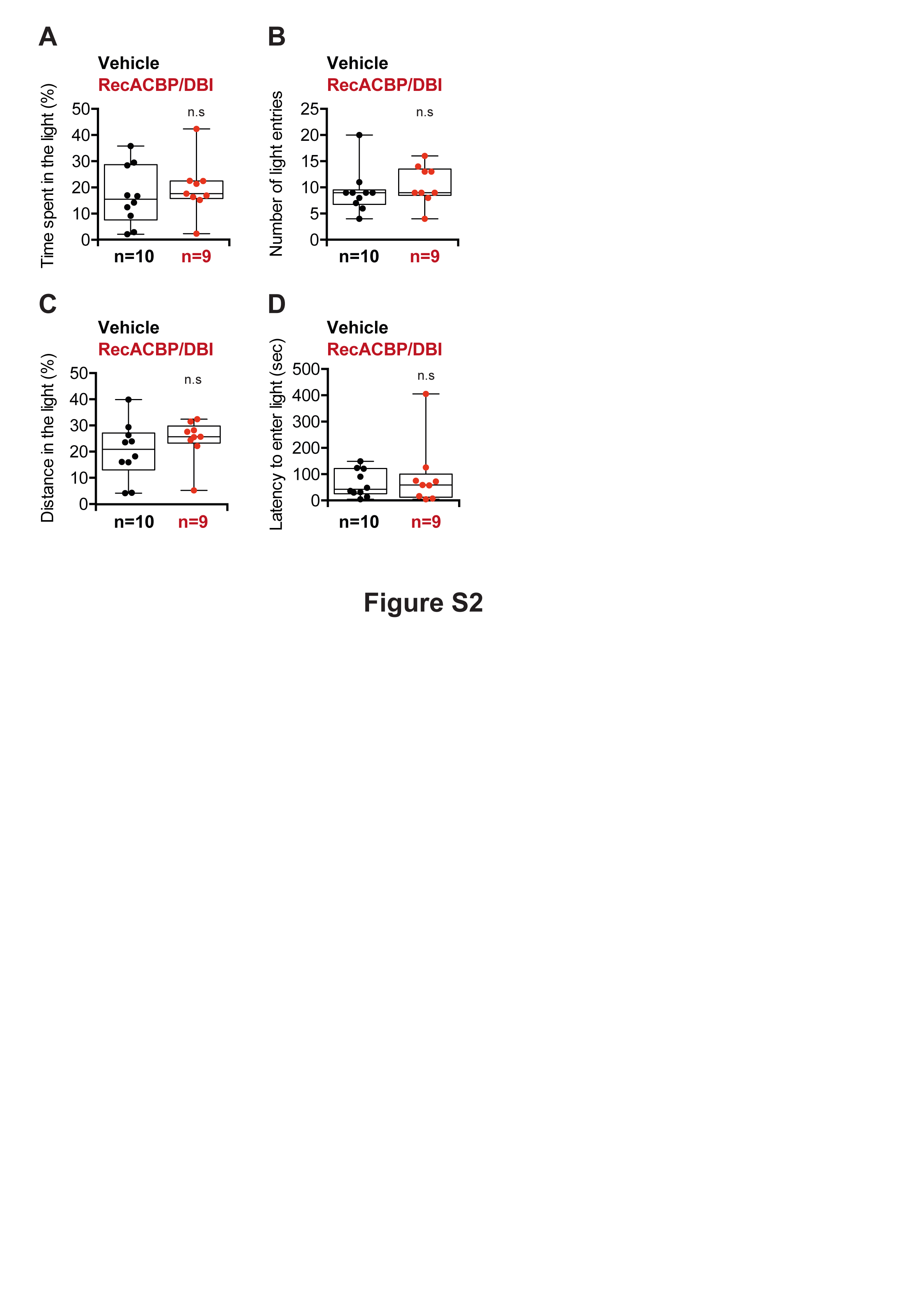

Supplement: Supplementary file 3 — Supplementary Figure S2 [file 41419_2020_2716_MOESM3_ESM.tif]

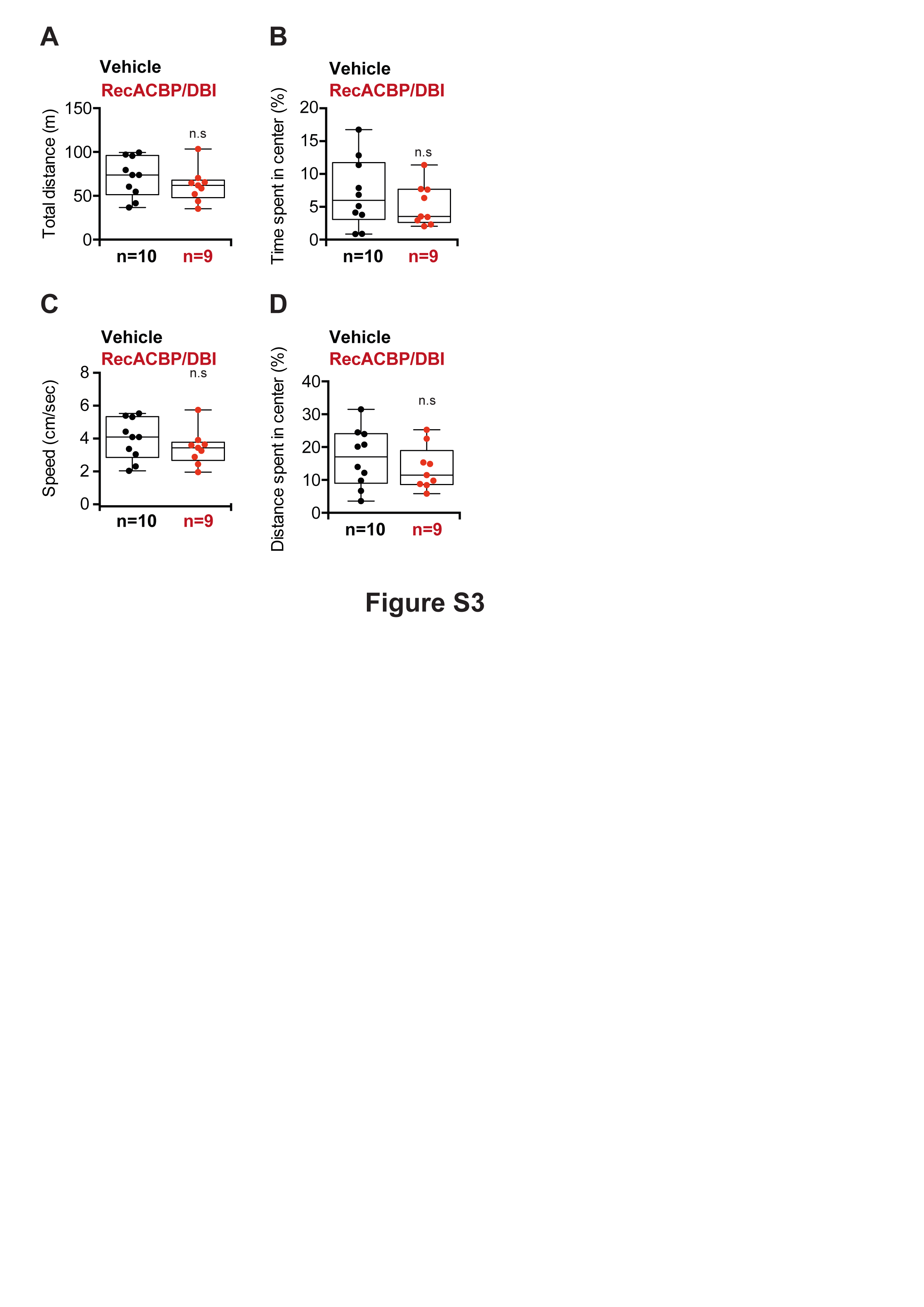

Supplement: Supplementary file 4 — Supplementary Figure S3 [file 41419_2020_2716_MOESM4_ESM.tif]

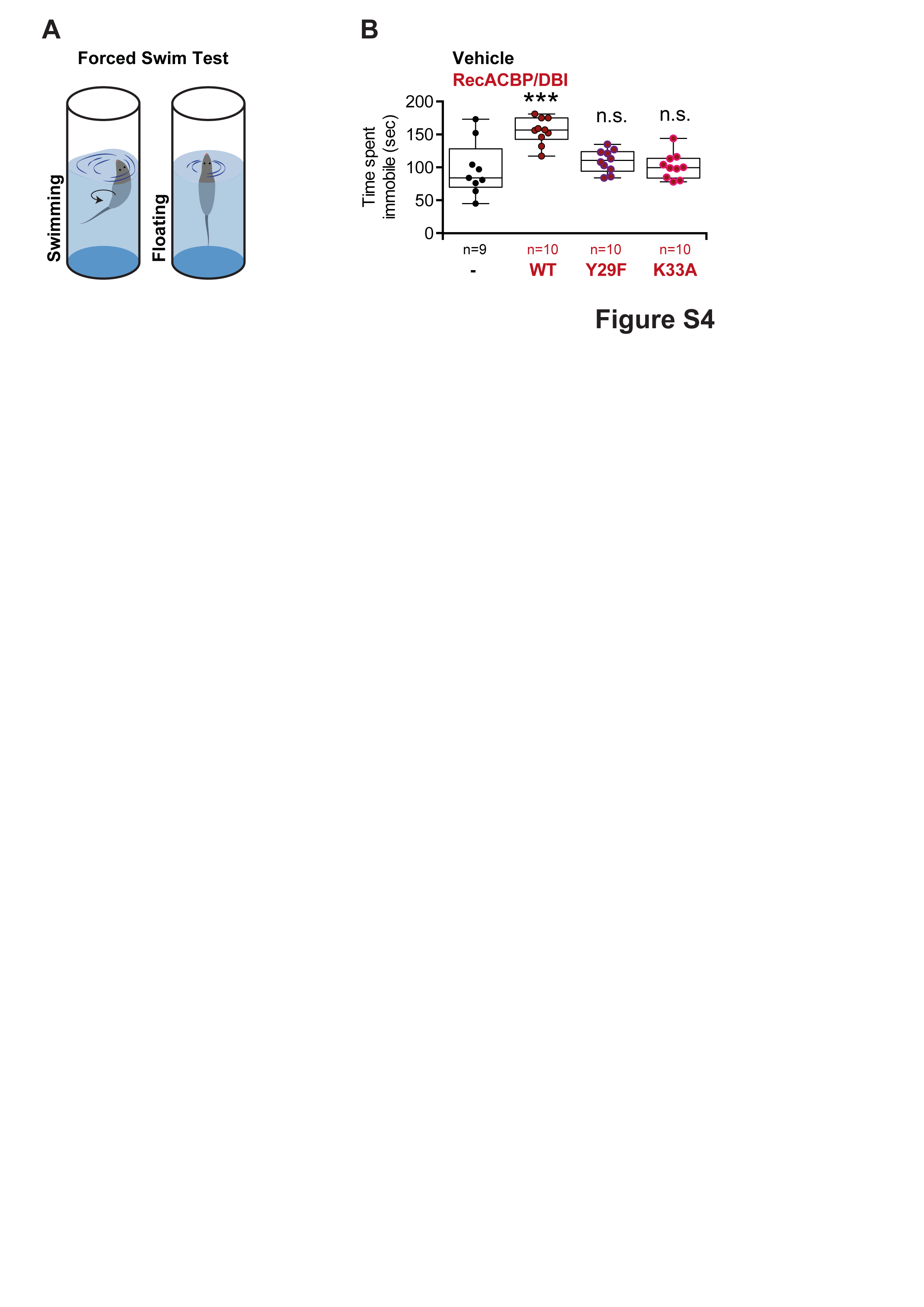

Supplement: Supplementary file 5 — Supplementary Figure S4 [file 41419_2020_2716_MOESM5_ESM.tif]

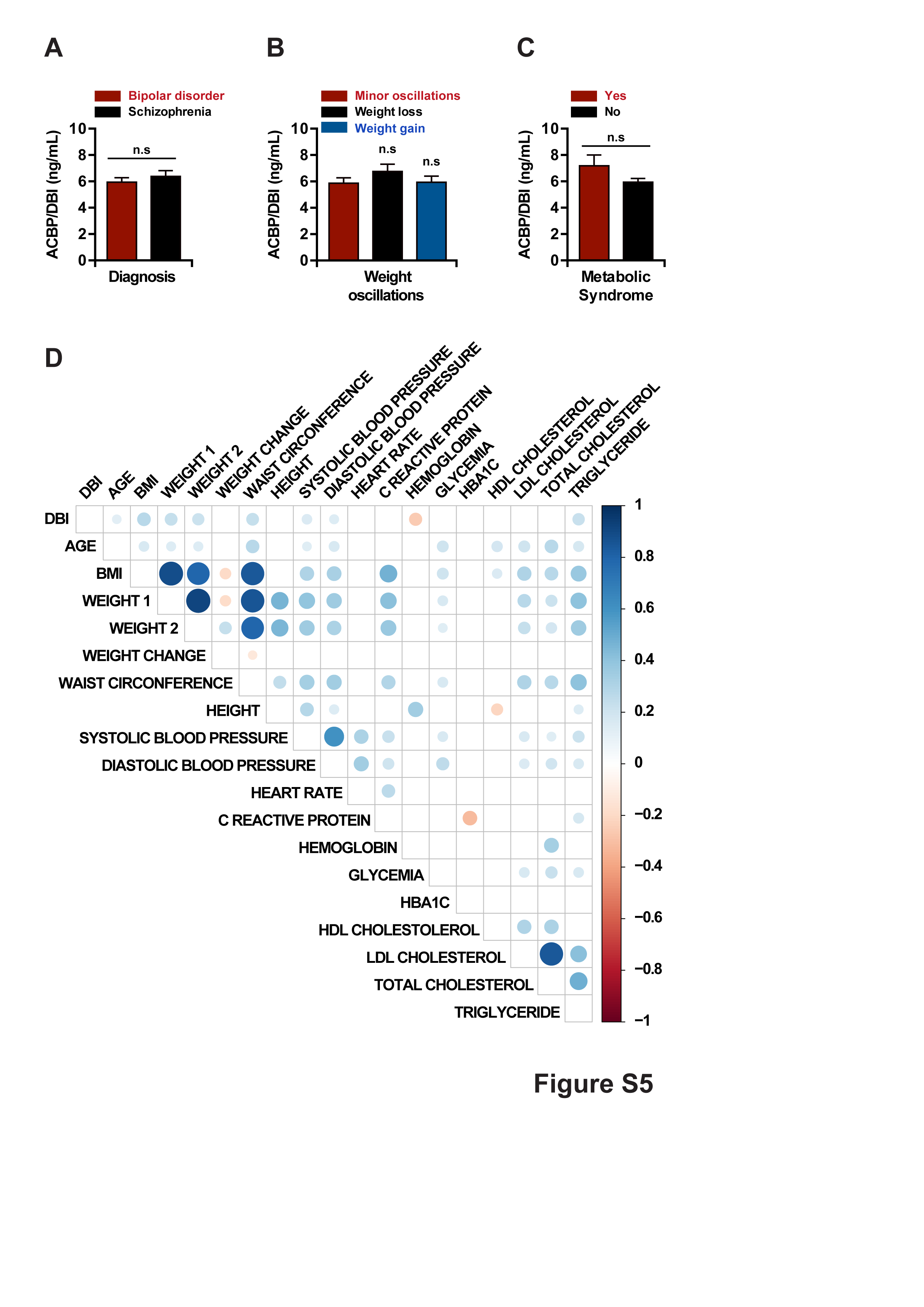

Supplement: Supplementary file 6 — Supplementary Figure S5 [file 41419_2020_2716_MOESM6_ESM.tif]
